# Supplementary material for: Ribosome profiling reveals ribosome stalling on tryptophan codons and ribosome queuing upon oxidative stress in fission yeast
Source: Nucleic Acids Res. 2020 Dec 11;49(1):383–99. doi: 10.1093/nar/gkaa1180 (PMC7797079; doi:10.1093/nar/gkaa1180)
Supplement: gkaa1180_Supplemental_Files [file gkaa1180_supplemental_files.zip › Supplementary_Material_Rubioetal_2020.pdf]

Table S1: Strain and experiment list

Genotype	Origin	Reference	Usage
972 h-	Lab collection	JU96	Ribo-seq, RNA-seq, drop assay, eIF2α phosphorylation
eIF2α-S52A::ura4$^+$ h-	Sandra *López-Avilés*	JU1502	Ribo-seq, eIF2α phosphorylation
sty1::ura4$^+$ ura4D18 h-	Sandra *López-Avilés, crossed out markers*	JU1503	Ribo-seq, eIF2α phosphorylation
fil1::kanMX6 h-	Lab collection	JU1594	RNA-seq, drop assay
fil1-TAP h+	Lab collection	JU1525	Fil1 expression
fil1-TAP eIF2α-S52A::ura4$^+$ h-	This work	JU1865	Fil1 expression
fil1-TAP sty1::ura4$^+$ h-	This work	JU1749	Fil1 expression

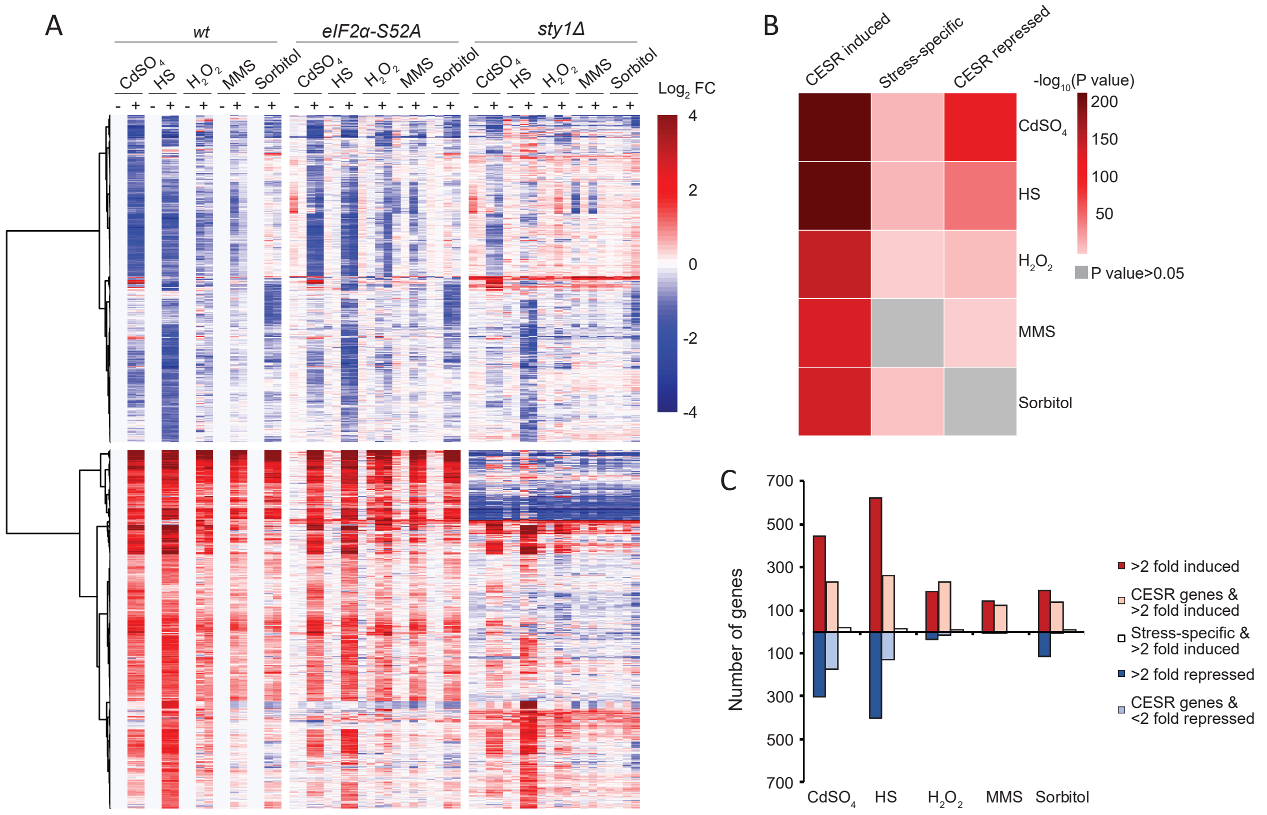

Supplementary Figure S1. Transcriptomic responses to stress. **(A)** Heat map of changes in mRNA levels in response to five stress conditions ($\log_2$ ratio stress/control). Data are shown for 1,247 genes that are differentially expressed in at least one stress in wild type (see Methods for details). All data are normalised to the corresponding untreated wild type sample. **(B)** Heat map displaying enrichment analysis of three published gene lists (CESR-induced, CESR-repressed and stress-specific genes) (14), and genes differentially expressed during five stress treatments (our data, see Supplementary Dataset S3). The overlap between each pair of gene lists (3x5 comparisons) was quantified, and the significance of the overlap calculated as described in Methods. **(C)** Comparison of the absolute numbers of induced and repressed genes in our experiments (>2-fold repressed or induced) and the overlap with the CESR-induced, CESR-repressed and stress-specific genes (14).

A

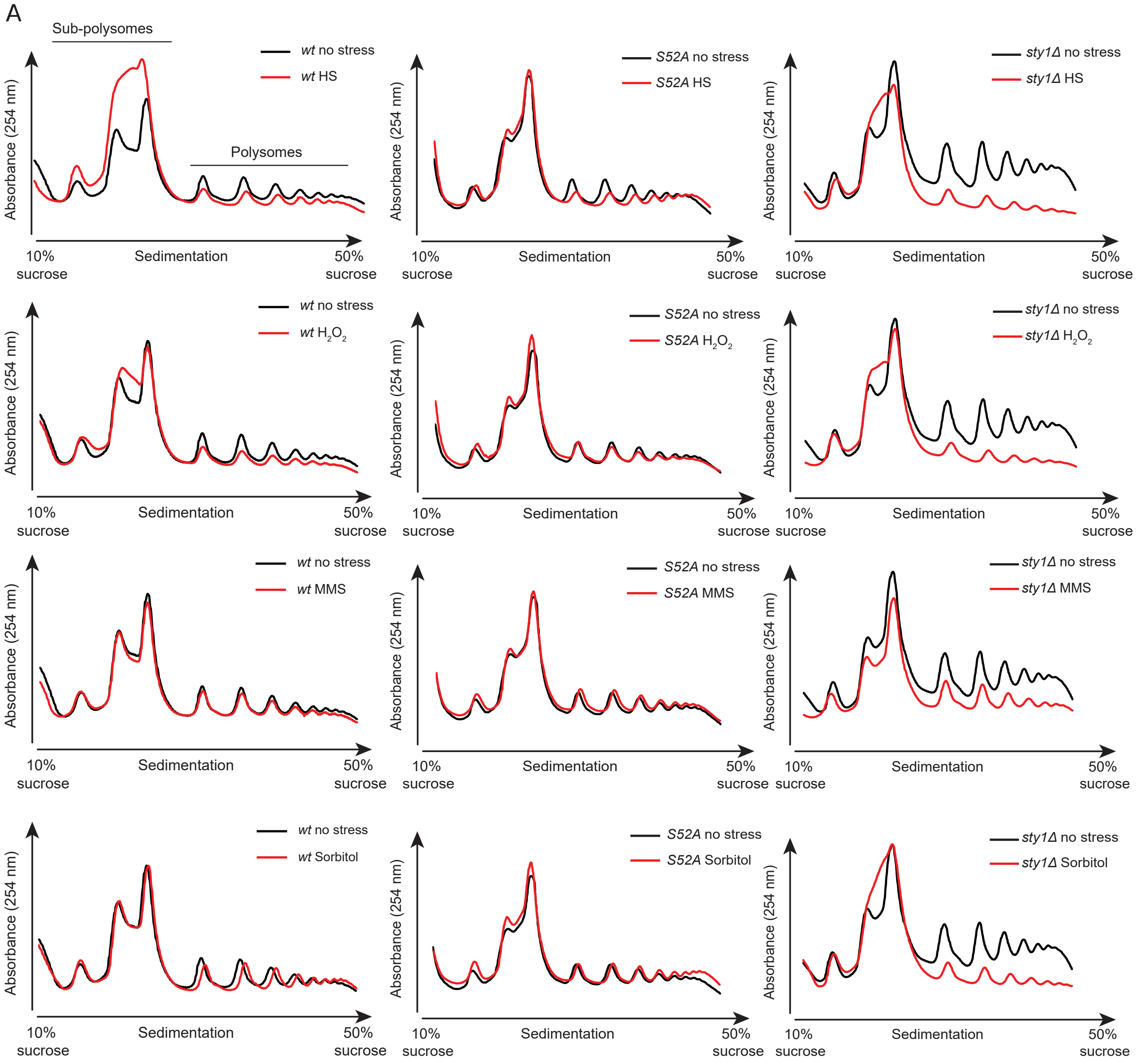

B

Polysomes/ Sub-polysomes ratios

	No stress	$CdSO_4$	HS	H_2O_2	MMS	Sorbitol
wt	0.20	0.07	0.07	0.10	0.18	0.18
	0.23	0.09	0.08	0.11	0.20	0.16
eIF2α-S52A	0.17	0.13	0.09	0.12	0.17	0.14
	0.22	0.11	0.09	0.17	0.22	0.15
sty1Δ	0.31	0.09	0.08	0.07	0.19	0.09
	0.28	0.11	0.07	0.05	0.24	0.06

C

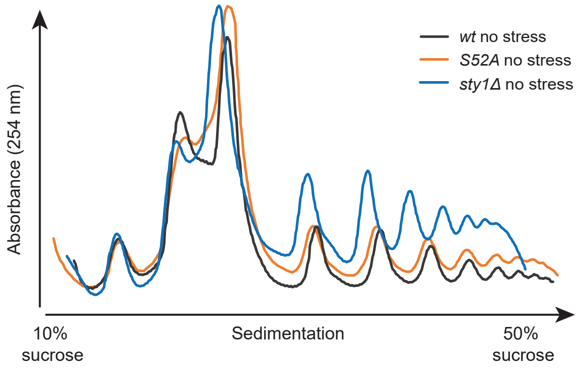

Supplementary Figure S2. General responses to stress. (A) Representative polysome profile traces before and after heat shock, H_2O_2 treatment, MMS exposure, and sorbitol treatment in wild type, *eIF2α-S52A* and *sty1Δ* cells. (B) Quantification of polysomes to subpolysomes ratios after five stress treatments (15 min). **(C)** As in A, directly comparing wild type, *eIF2α-S52A* and *sty1Δ* cells in unstressed conditions.

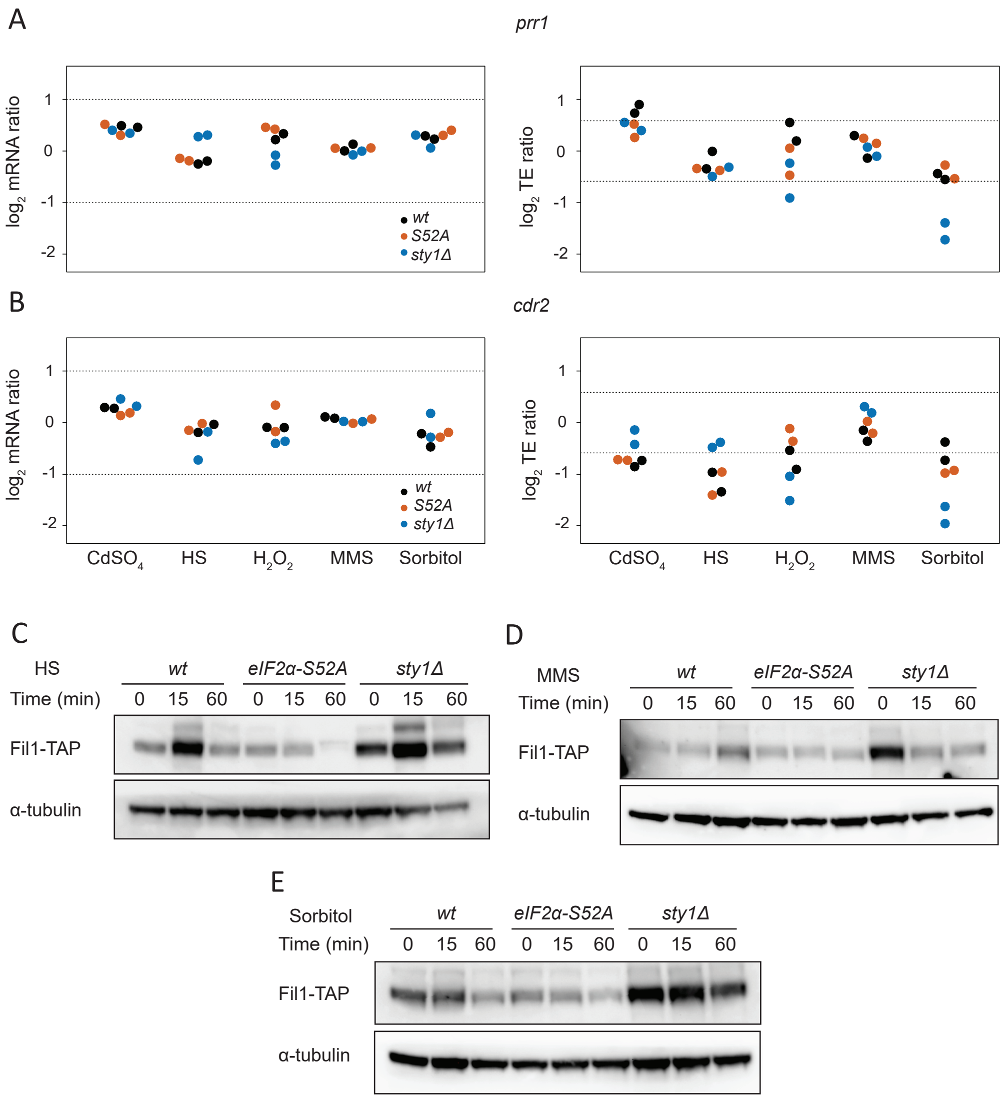

Supplementary Figure S3. Gene-specific translational regulation. **(A)** Changes in translation efficiency and transcript levels (log_2 ratios stress/control) for the *prr1* gene. Data for two biological replicates are shown. **(B)** As in A, but for the *cdr2* gene. **(C to E)** Western blots to measure Fil1-TAP protein levels after heat shock, MMS and sorbitol at the indicated times and genetic backgrounds. Tubulin was used as a loading control.

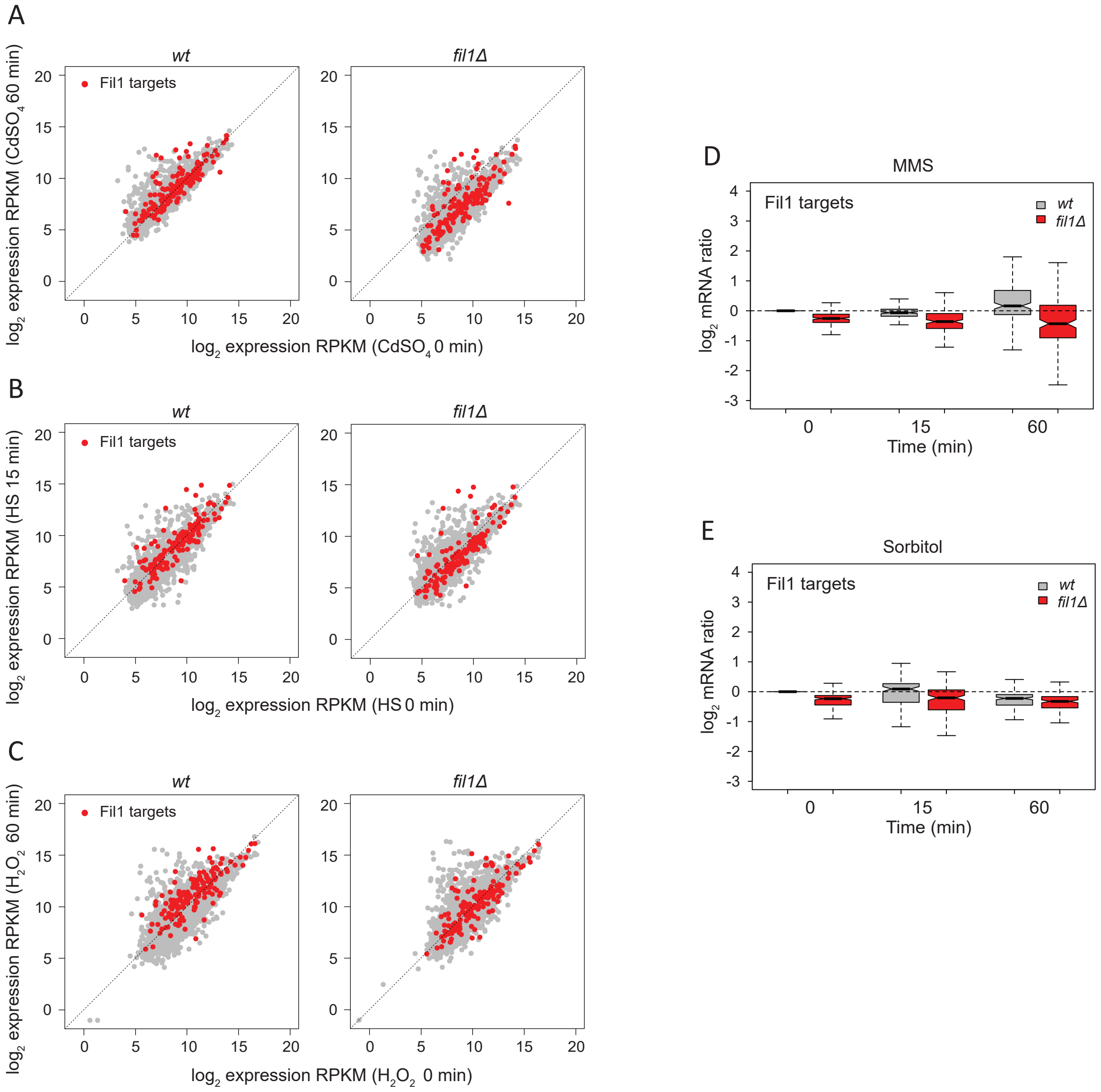

Supplementary Figure S4. Expression of Fil1 targets in *fil1Δ* mutants under stress conditions. (A to C) Scatter plots comparing mRNA levels of stressed and control cells (log_2 RPKM) in wild type and *fil1*Δ cells at the indicated times and stresses. Fil1 targets are highlighted in red. **(D, E)** Boxplot showing changes in mRNA levels (log_2 ratio stress/control) of genes encoding Fil1 targets after MMS and sorbitol treatments, in wild type and *fil1Δ* strains.

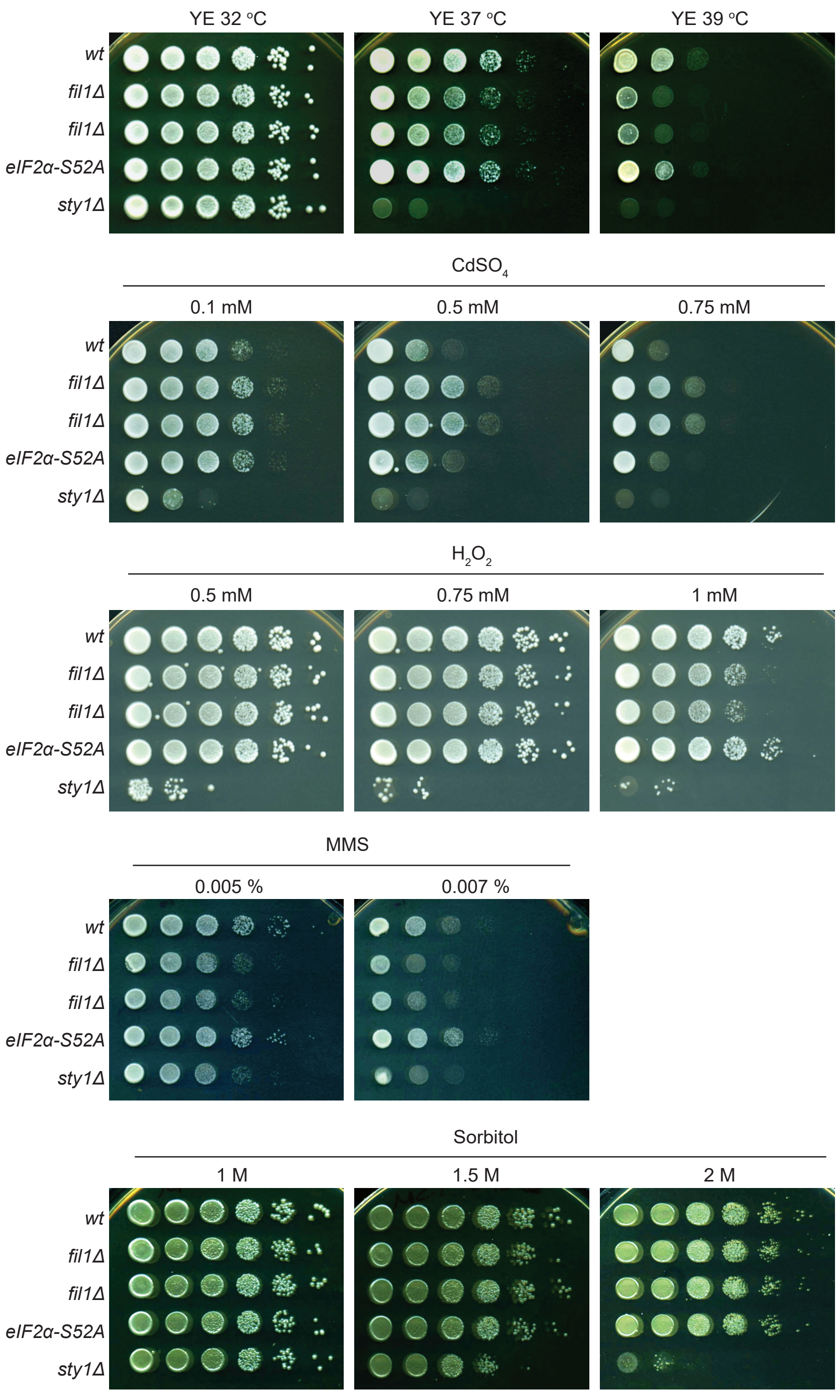

Supplementary Figure S5. Cell viability assays. Drop assays of wild type, *fil1Δ*, *eIF2α-S52A* and *sty1Δ* cells plated in the indicated conditions.

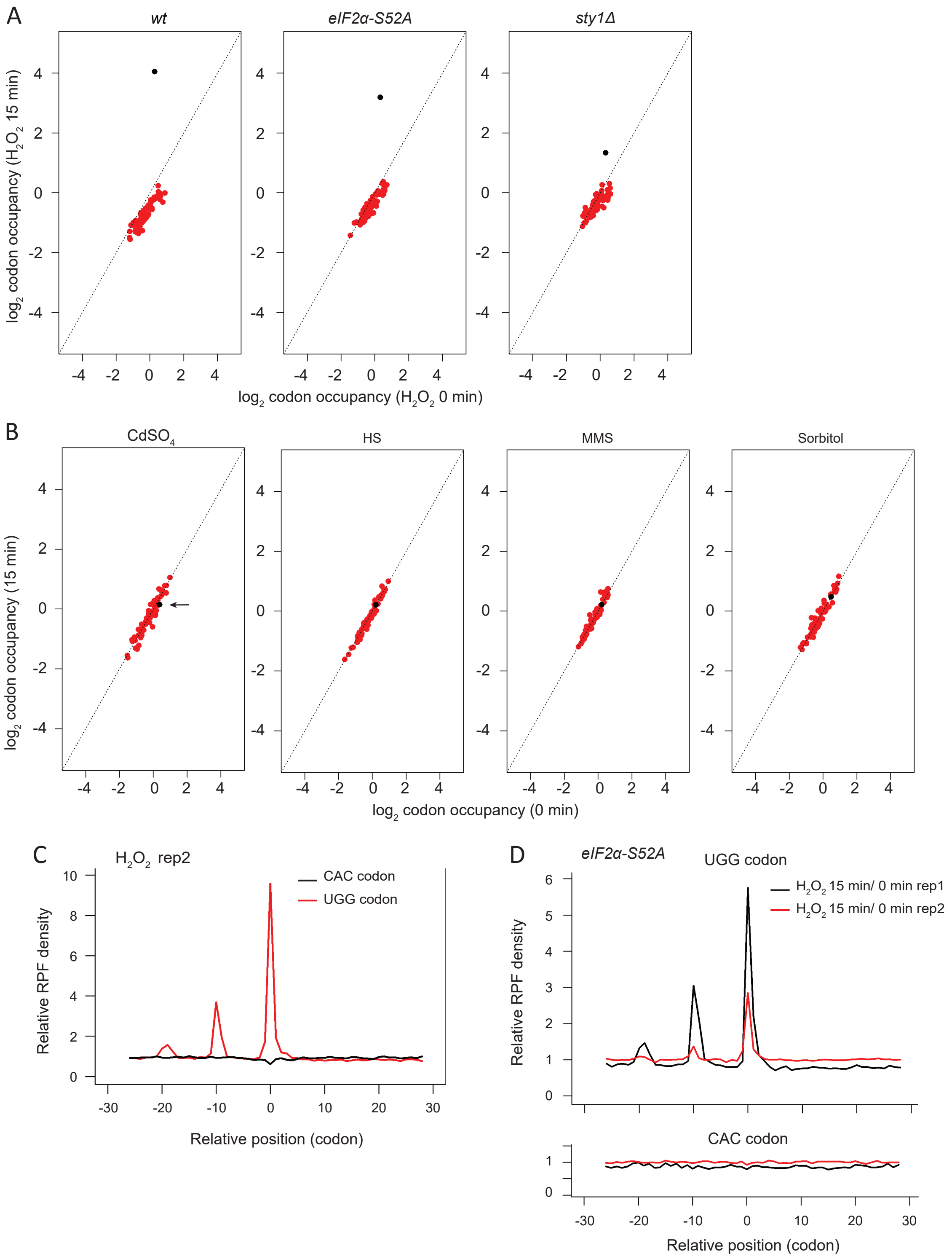

Supplementary Figure S6. Tryptophan codon enrichment is specific for H_2O_2 treatment. (A) Scatter plots showing log_2 relative codon occupancies before and after H_2O_2 treatment for 15 minutes in wild type cells (as in Figure 5A, but a different biological replicate), in *eIF2α-S52A*, and in *sty1Δ* strains. The dot corresponding to the UGG codon, encoding tryptophan, is shown in black. **(B)** As in A, for wild type after cadmium, heat shock, MMS and sorbitol treatments. UGG codon is displayed in black and highlighted with an arrow. **(C)** Metagene depicting average read density of RPFs around tryptophan codons (UGG) or one of the histidine codons (CAC) after H_2O_2 treatment (as in Figure 5B, but a different biological replicate). **(D)** As in C, but in two different biological replicates in *eIF2α-S52A* cells.

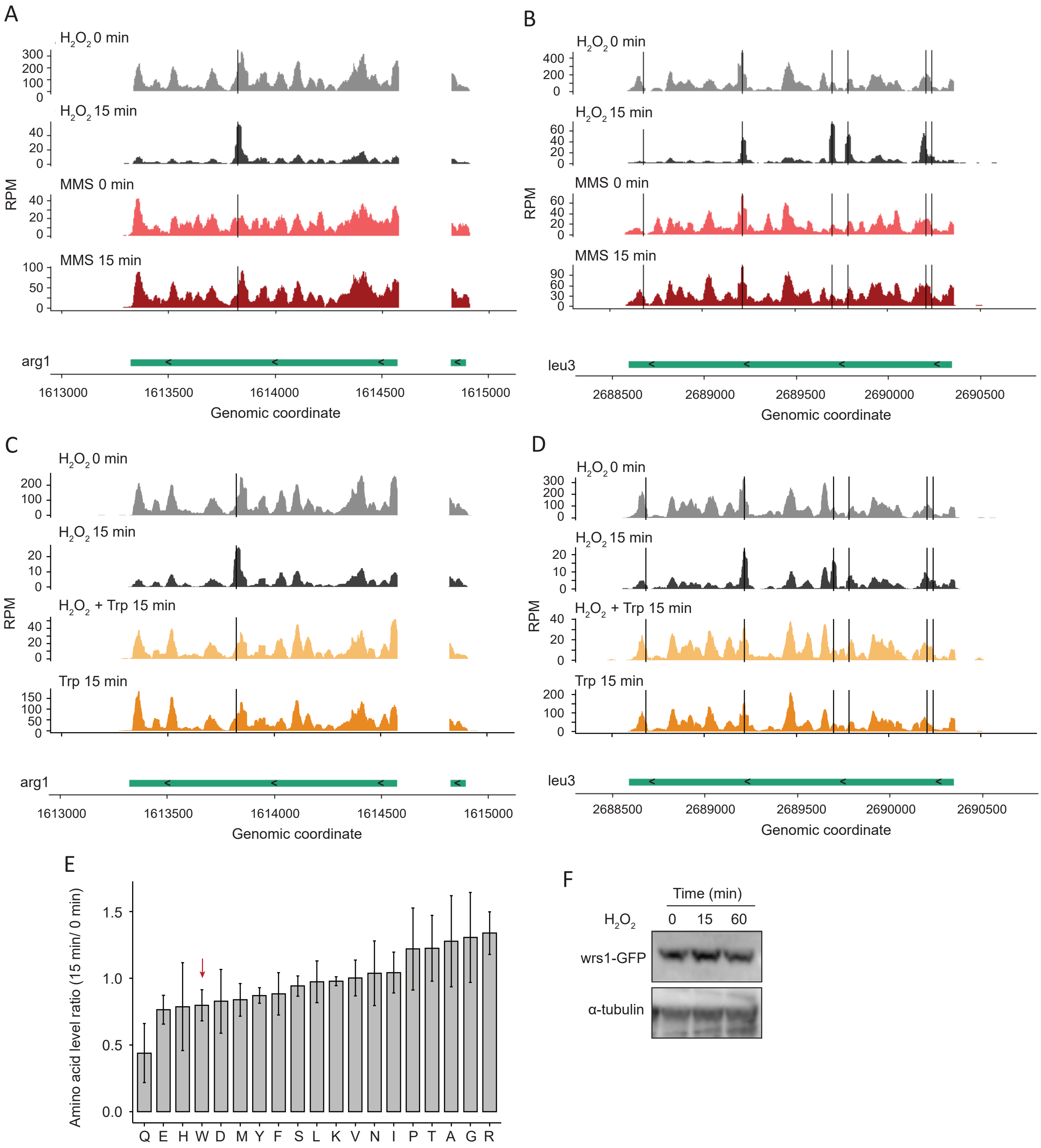

Supplementary Figure S7. Ribosome stalling on tryptophan codons. (A-B) Profiles of RPF coverage normalized to the number of aligned reads per million (RPM) for the indicated genomic regions (corresponding to the *arg1* and *leu3* genes) in control cells and after H_2O_2 and MMS treatments. The majority of the UGG codon positions, indicated by black lines (1 codon in *arg1* and 6 codons in *leu3*), showed ribosome stalling. **(C-D)** As in A and B, but for control cells, H_2O_2 treatment, H_2O_2 and tryptophan together, and tryptophan-only addition to the medium. **(E)** Change in amino acid levels (ratio stress/control) upon oxidative stress exposure. Data are from 4 independent biological replicates (means ± SD). Tryptophan (W) is highlighted by a red arrow. **(F)** Western blot analysis of Wrs1::GFP (cytoplasmic tryptophan-tRNA ligase) levels before and after H_2O_2 treatment. Tubulin was used as a loading control.

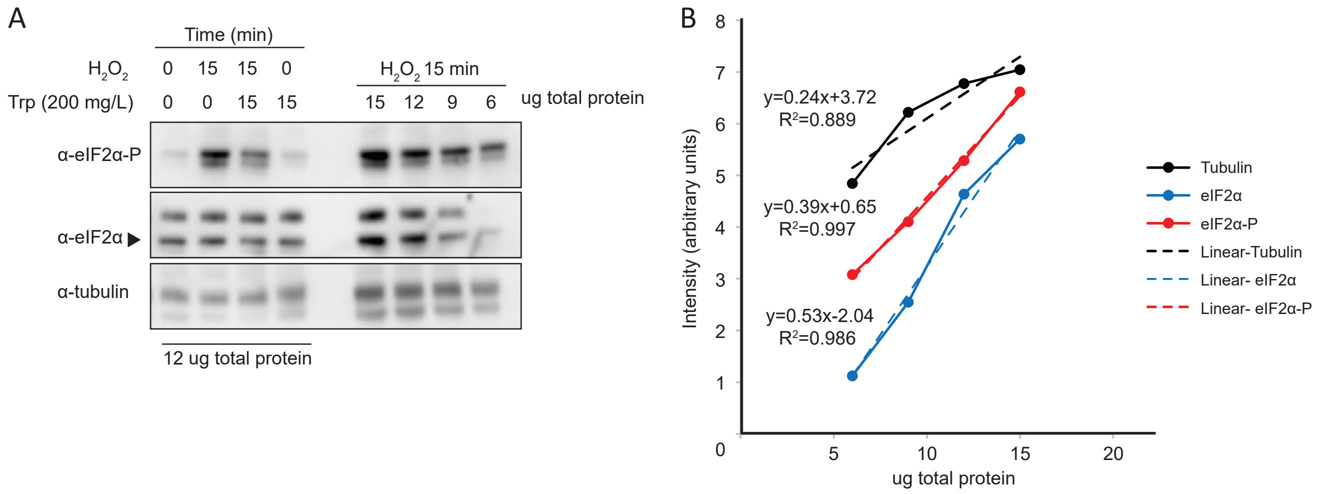

Supplementary Figure S8. Antibody linearity quantification. **(A)** Representative western blot quantified in Figure 6B showing a dilution series of the second lane sample. **(B)** Graph displaying intensity measurements of protein dilutions of a representative western blot using the antibodies against eIF2α-P and eIF2α total under the conditions of the experiments in Figure 6A. R-square and equation values are shown for each antibody.

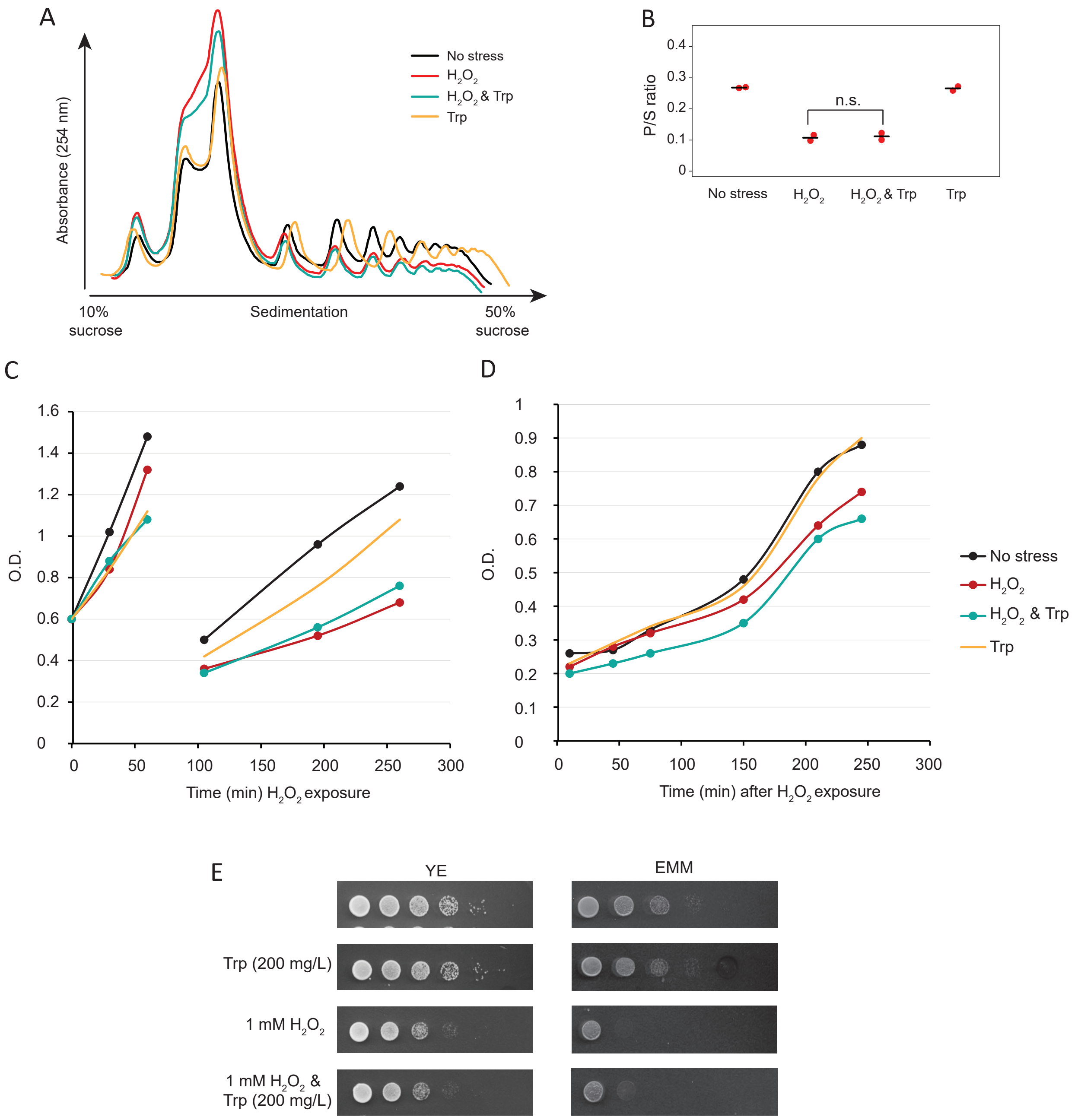

Supplementary Figure S9. Addition of tryptophan during H_2O_2 treatment does not affect polysomes to subpolysomes ratios, growth or viability. **(A)** Polysome profile traces in wild type cells treated as indicated (0.5 mM H_2O_2 and/or 200 mg/L tryptophan as required) for 15 minutes. **(B)** Quantification of polysomes to subpolysomes ratios after the treatments in A. The data are shown for two independent biological replicates. **(C)** Graph representing growth (optical density) in cells subjected to the treatments as in A. Note there is a dilution gap to extend the treatment under exponential growth. **(D)** As in C, but cells were exposed to 0.5 mM H_2O_2 for 260 minutes, washed and released into the indicated conditions. **(E)** Drop assay of wild type cells plated in the indicated conditions.

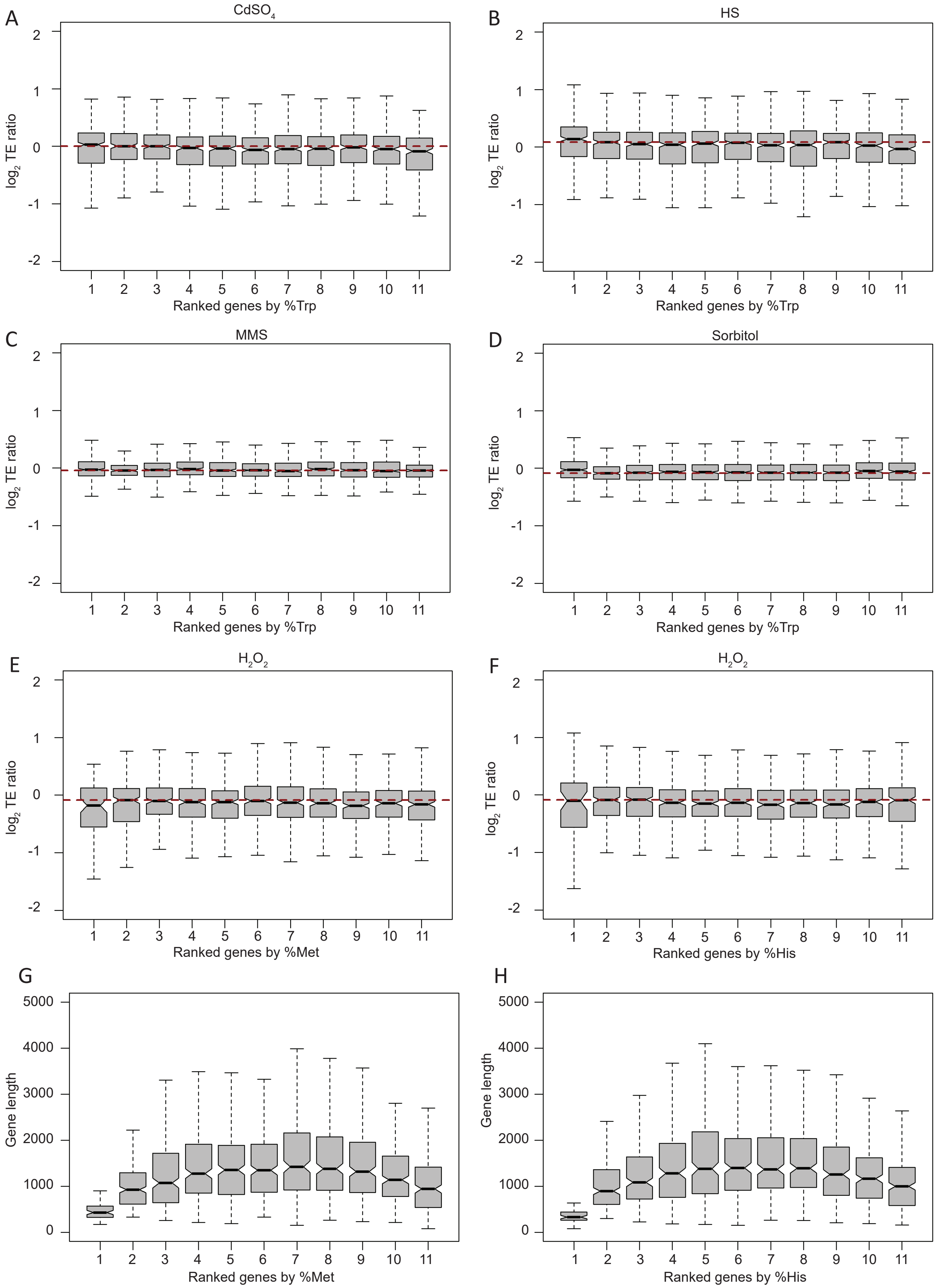

A
CdSO4
B
HS
C
MMS
D
Sorbitol
E
H2O2
F
H2O2
G
H
log2 TE ratio
Gene length
Ranked genes by %Trp
Ranked genes by %Met
Ranked genes by %His

Supplementary Figure S10. Translation efficiency of tryptophan codon-enriched genes after other stress treatments, and of other codons upon H_2O_2 treatment. **(A** to **D)** Boxplots displaying changes in translation efficiency upon the indicated stresses ($\log_2$ stress/control ratios) according to tryptophan codon content. Genes were binned into 11 categories based on the fraction of tryptophan codons in their coding sequences (the first group contains 269 genes lacking tryptophan codons, and the other 10 groups have 234 genes each). The horizontal red dashed line indicates the median of the second group. **(E** and **F)** Similar analyses to A-D, but for methionine and histidine codons. **(G** and **H)** As above, but displaying coding sequence lengths.
